# Spatial-Division Multiplexing Approach for Simultaneous Detection of Fiber-Optic Ball Resonator Sensors: Applications for Refractometers and Biosensors

**DOI:** 10.3390/bios12111007

**Published:** 2022-11-11

**Authors:** Madina Shaimerdenova, Takhmina Ayupova, Aliya Bekmurzayeva, Marzhan Sypabekova, Zhannat Ashikbayeva, Daniele Tosi

**Affiliations:** 1 School of Engineering and Digital Sciences, Nazarbayev University, 53 Kabanbay Batyr, Astana 010000, Kazakhstan; 2 Department of Bioengineering and Nick Holonyak Micro and Nanotechnology Laboratory, University of Illinois at Urbana-Champaign, Urbana, IL 61801, USA; 3 National Laboratory Astana, Laboratory of Biosensors and Bioinstruments, 53 Kabanbay Batyr, Astana 010000, Kazakhstan; 4 School of Engineering and Computer Science, Baylor University, Waco, TX 76798, USA

**Keywords:** optical-fiber sensors, refractive index sensors, multiplexing, spatial-division multiplexing, ball resonators, fiber-tip sensors, cancer biomarkers, biosensors multiplexing

## Abstract

Fiber-optic ball resonators are an attractive technology for refractive index (RI) sensing and optical biosensing, as they have good sensitivity and allow for a rapid and repeatable manufacturing process. An important feature for modern biosensing devices is the multiplexing capacity, which allows for interrogating multiple sensors (potentially, with different functionalization methods) simultaneously, by a single analyzer. In this work, we report a multiplexing method for ball resonators, which is based on a spatial-division multiplexing approach. The method is validated on four ball resonator devices, experimentally evaluating both the cross-talk and the spectral shape influence of one sensor on another. We show that the multiplexing approach is highly efficient and that a sensing network with an arbitrary number of ball resonators can be designed with reasonable penalties for the sensing capabilities. Furthermore, we validate this concept in a four-sensor multiplexing configuration, for the simultaneous detection of two different cancer biomarkers across a widespread range of concentrations.

## 1. Introduction

Optical-fiber refractometers are an emerging technology, which allows for the realization of compact sensing devices with a miniature footprint, achieving high performance, compatibility, and overall superiority with respect to optical refractometers [1]; in general, optical refractometers are either operated as a benchtop or portable instrument, particularly when using plasmonic devices [2], while optical-fiber devices can operate in situ and perform localized detections [3]. Optical-fiber refractometers measure the refractive index (RI) changes occurring in the medium surrounding the fiber sensor. This approach plays an important role in the monitoring of contaminations, for example, by estimating the water quality with a real-time system [4]. However, most notably, RI sensors are the building blocks of optical biosensors [5], converting the refractometer structure into a selective detector of biological analytes through a surface-functionalization process. Optical-fiber biosensors are important tools for immunosensing [6] and for the detection of cardiac [7] and cancer biomarkers [3], among others.

Optical-fiber sensors are inherently suitable for multiplexing (sensing networks that can encompass hundreds of sensors) [8] and distributed sensing (sensing networks that enable continuous, real-time measurements along the entire length of a fiber-optic cable) [9]. Several methods have been proposed for physical sensors based on multiplexed sensing. Such sensors have been used for temperature, pressure, and strain sensing [10,11]. Multiplexed sensing networks can be classified into the following subgroups: time-division multiplexing (TDM), wavelength-division multiplexing (WDM), polarization-division multiplexing (PDM), spatial-division multiplexing (SDM), and cepstrum-division multiplexing (CDM).

TDM uses a switch to commute between different channels; it is a simple architecture that is effective in most sensing networks, but it requires the introduction of an additional component (the switch, with an electrical controller), and, in large networks, it slows down the data acquisition rate, hence losing the simultaneous detection of multiple sensors. WDM is an effective method with fiber Bragg gratings (FBGs), which are narrowband sensors [12]; inline sensors are multiplexed by assigning a different wavelength slot to each element of an FBG array. PDM, common in telecommunication links [13], does not achieve great impact in sensing networks, as it allows for only a maximum of a 2× channel increase.

In recent years, emerging methods for multiplexing have been implemented, having a much more specific implementation, which are designed for a particular type of sensor or for specific hardware. SDM has been implemented in multicore fibers, with the capability of resolving inline FBGs [14] and Fabry–Perot interferometers [15], and allows for implementing up to three-dimensional-shape sensing with a single fiber [16]. CDM has been reported for broadband sensors with a quasi-periodic spectrum, such as interferometers [17]. More recently, methods based on scattering-level multiplexing have been reported in order to simultaneously detect distributed fiber links, achieving three-dimensional-shape [18] and temperature [10] sensing.

While multiplexing has been mainly reported for physical sensors, it has also achieved a significant impact in biological and biomedical sensing: in the framework of real-time RI sensing and biosensing, multiplexing plays a significant role, particularly when looking at the integration between the sensing device and the application [19]. Chen et al. developed a multiplex immunoassay for detection of serum cytokines using a localized surface plasmon resonance-based biosensor (LSPR) and stated that the technique allowed them to measure cytokine concentrations from 5 to 20 pg/mL in a 1 μL serum sample [20]. Another simultaneous detection of ovarian and breast cancer markers using a photonic crystal surface mode for real-time analyte ligand monitoring was described by Petrova et al. In the above method, the limit of detection for the human epidermal growth factor receptor 2 (HER2) biomarker was 620 fg/mL, with a linearity range of up to 50 pg/mL, and the minimal detectable concentration for biomarkers CA15-3 and CA125 was 1.84 U/mL and 0.55 U/mL, respectively [21].

Multiplexing techniques, however, have seldom been applied to fiber-optic RI sensors and biosensors, due to their disadvantageous spectral properties that hamper WDM and similar methods. Some techniques for the multiplexing of RI sensors are based on scattering-level multiplexing for multiple readouts of reflector-less sensors [22] or based on the use of broadband optical spectral analyzers to simultaneously detect a pair of surface plasmon resonance (SPR) inline sensors [19].

In this work, simultaneous detection of two cancer biomarkers, namely CD44 (a cell-surface glycoprotein) and HER2 (human epidermal growth factor receptor-2), has been performed. HER2 (185 kDa) is part of the human epidermal growth factor receptor family, which participates in cell growth, proliferation, and differentiation. It is a transmembrane protein that has three domains: an intracellular tyrosine kinase domain, a transmembrane lipophilic segment, and an extracellular domain (ECD). HER2 can be activated by (1) dimerizing with another HER2 family member or (2) proteolytic cleavage of its ECD. The activation of HER2 triggers the downstream activation of multiple signaling pathways. HER2 is usually expressed at low levels (15–75 ng/mL) by epithelial cells of the breast, lung, kidney, ovary, and other tissues [23,24,25,26]. HER2 overexpression is associated with the occurrence and progression of aggressive types of breast cancer. The cleavage of the ECD domain from HER2 during breast cancer significantly facilitates tyrosine kinase activity. The formation of a truncated receptor after the cleavage increases the oncogenicity by 10–100-fold compared to the untruncated protein [27]. The shedding of ECD is usually associated with tumor metastasis. The detection of ECD (or soluble HER2) in serum is particularly relevant for the diagnosis of cancer recurrence and metastasis [28].

A small number of cells, called cancer stem cells (CSC), are thought to play a major role in the recurrence, metastasis, and resistance to therapy observed in cancer [29]. CD44, or cluster differentiation 44, is one of the most well-known CSC markers. It is a surface glycoprotein that is part of the surface adhesion molecules [30]. Increased expression and dysregulation of this protein are associated with the initiation and progression of cancer, playing an important role in tumor metastasis and developing resistance to therapy [31]. A recent meta-analysis, which included 48 studies, revealed that, in colorectal cancer, increased CD44 could be utilized as prognostic factor and is specifically correlated with such parameters as poor differentiation and metastasis of the lymph nodes and distant organs [32]. CD44 is also present in a soluble form in different biological fluids including serum. In human malignancies, serum CD44 has been found to be increased, and it was correlated with poor prognosis and metastasis [33]. In breast cancer, the protein was more increased in patients with metastasis in the liver than in patients with metastasis to other organs [30]. Evaluation of serum CD44 before surgery in oral squamous cell carcinoma was found to be a reliable way of differentiating patients with a high risk of recurrence from those without it [34].

In this work, we introduce an SDM method for the simultaneous interrogation of multiple optical-fiber ball resonators (BR) [35]. BR sensors represent a good alternative to refractometers and biosensors based on gratings or plasmonic principles [6]. BRs have much simpler and rapid fabrication process, which is based on well-known concepts in the fabrication of diffractive lenses [36]. BR sensors yield a quasi-random spectral fingerprint, with weak finesse and can be interrogated by analyzing changes of amplitude in the spectral fringes [37], or a wavelength shift in case of more identifiable spectral envelopes [38].

Because of their inherent weak reflectivity and low finesse, BR sensors are suitable for being detected only by interrogators operating in reflection mode and capable of resolving very low power levels, such as the optical backscatter reflectometer (OBR) [39]. Thanks to the spatial distribution features [9], we design an SDM method that allows for connecting and simultaneously scanning an arbitrary number of BR sensors. The SDM approach solves the problem of enabling multiplexing specifically for BR sensors, since they appear as wideband shallow sensors, and, therefore, neither wavelength-based methods nor cepstrum- or other transform-based methods would work, since BRs share the same bandwidth and have a non-periodical spectral envelope.

Besides reporting the SDM protocol for multiplexing BR sensors, we evaluate the performance of the system in a four-sensor configuration, by comparing the sensitivity levels of individual and multiplexed BRs, evaluating the cross-talk, and showing the effect of the multiplexing setup on the spectra of the BRs. Then, we prove the multiplexing technique can be applied to a biosensing application, employing four sensors with two different biofunctionalizations for the simultaneous detection of two cancer biomarkers, namely CD44 [30] and HER2 [23], which are both employed in the diagnostics of breast cancer diseases [27,31].

## 2. Materials and Methods

### 2.1. Reagents

The (3-Aminopropyl) trimet hoxysilane (APTMS), glutaraldehyde (GA), phosphate-buffered saline (PBS), hydrogen peroxide (H_2_O_2_), methanol, human serum, and sulfuric acid (H_2_SO_4_) were purchased from Sigma-Aldrich (Darmstadt, Germany). Bovine serum albumin (BSA) and poly(ethylene glycol) methyl ether amine (mPEG-amine) were obtained from Thermo Fisher Scientific (Runcorn, UK) and Sigma-Aldrich respectively. Human ErbB2/HER2 (Research Grade Trastuzumab Biosimilar) antibody (MAB9589-100) and recombinant human ErbB2/HER2 protein (10126-ER-050) were purchased from Bio-Techne Ltd. (Oxford, UK). CD44 monoclonal antibody (SAB4700179) was purchased from Sigma-Aldrich (Darmstadt, Germany), and recombinant human CD44 protein (Active) (ab173996) was purchased from Abcam (Cambridge, UK).

### 2.2. Fabrication of Ball Resonators

BRs were fabricated using CO_2_ laser (LZM-100 CO_2_, Fujikura Ltd., Tokyo, Japan). CO_2_ laser’s heat source is widely used for optical-fiber splicing and fabrication of numerous devices of different geometries for various applications. In this work, ball resonators were fabricated using a method described in [38]. As a result, four ball resonators of different sizes, 378–374 μm, 386–380 μm, 433–426 μm, and 449–443 μm, were fabricated with different parameters such as heating power, ball geometry, rotation, and feeding speed.

The fabrication of the devices follows the same process described in previous works [35]. By setting the hereby reported values for target diameter and motion speed, we can achieve a BR sensor that appears as a good trade-off between a spherical shape and sufficient back-reflected power, which enables the spectral interrogation. The obtained spectra of BR sensors each appear as a quasi-random spectrum with a dependence on the refractive index, as outlined in previous works [35,40].

**Figure 1:**
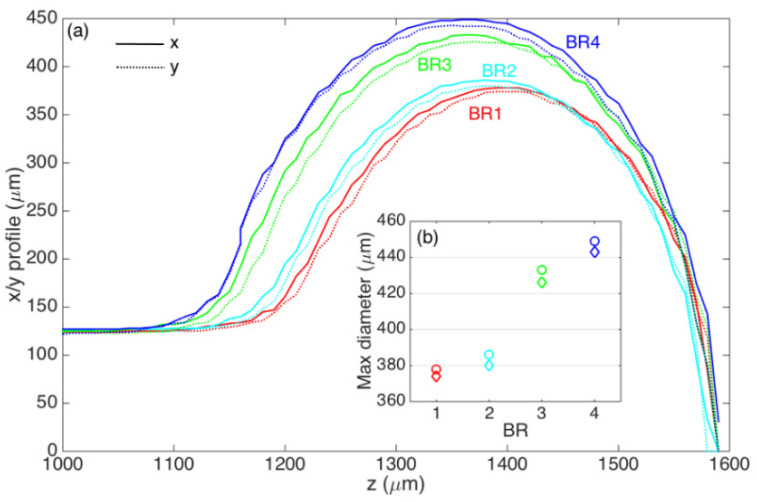
Profilometry of the four ball resonators fabricated with the CO_2_ laser splicer. (**a**) Profilometry chart, reporting the local diameter along x (solid lines) and y (dotted lines) along the fiber direction z, for each ball resonator BR1 (red), BR2 (cyan), BR3 (green), and BR4 (blue). (**b**) Maximum diameter for each ball resonator, evaluated along x (circles) and y (diamonds).

**Figure 2:**
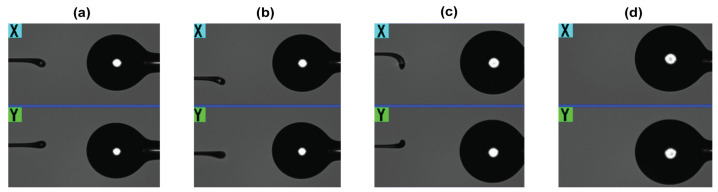
Ball resonator images taken by X and Y cameras of CO_2_ laser splicing system (**a**) 378–374 µm (BR1), (**b**) 386–380 µm (BR2), (**c**) 433–426 µm (BR3), (**d**) 449–443 µm (BR4). BRs are shown on the right side of the images, while the left part shows the second fiber used for the formation of the spherical device.

The profilometry of the four ball resonators is shown in Figure 1, which reports the local diameter along *x* and *y* directions for each location *z* along the fiber axis. The ball resonators have different *x*–*y* diameters, respectively, 378–374 μm (BR1), 386–380 μm (BR2), 433–426 μm (BR3), and 449–443 μm (BR4). All BR sensors have a profile slightly more elongated along the x direction; the ellipticity for the ball resonators, defined as e = [1 − (d_y_/d_x_)^2^]^1/2^, where d_x_, d_y_ are the maximum diameters along x and y, is equal to e = 0.145 (BR1), e = 0.176 (BR2), e = 0.179 (BR3), and e = 0.162 (BR4), respectively. Photographs of the ball resonators used in the experiments are shown in Figure 2.

### 2.3. Multiplexing Arrangement and Experimental Setups

The interrogator used in the experiments is an optical backscatter reflectometer (OBR 4600, Luna Inc., Roanoke, VA, USA), which implements the optical frequency domain reflectometry principle [39] with a detector capable of resolving low power rates, and, therefore, determining the spectrum of the weak reflective fringes of BRs. The multiplexing scheme is based on the capability of the OBR to resolve spectra in the frequency domain, with spatial resolution around 10 μm operating with a laser-scan window of 85 nm in the infrared [39]. Since all sensors have a broadband spectrum that makes it impossible to use a WDM-like approach, we encode the diversity parameter in the “space” where each sensor is read on the OBR, which is then implemented by adding a delay line to each element of the sensing network. This approach has been demonstrated using a commercial OBR, but in principle it can work with any optical-frequency-domain reflectometer having a scanning laser as light source [20].

**Figure 3:**
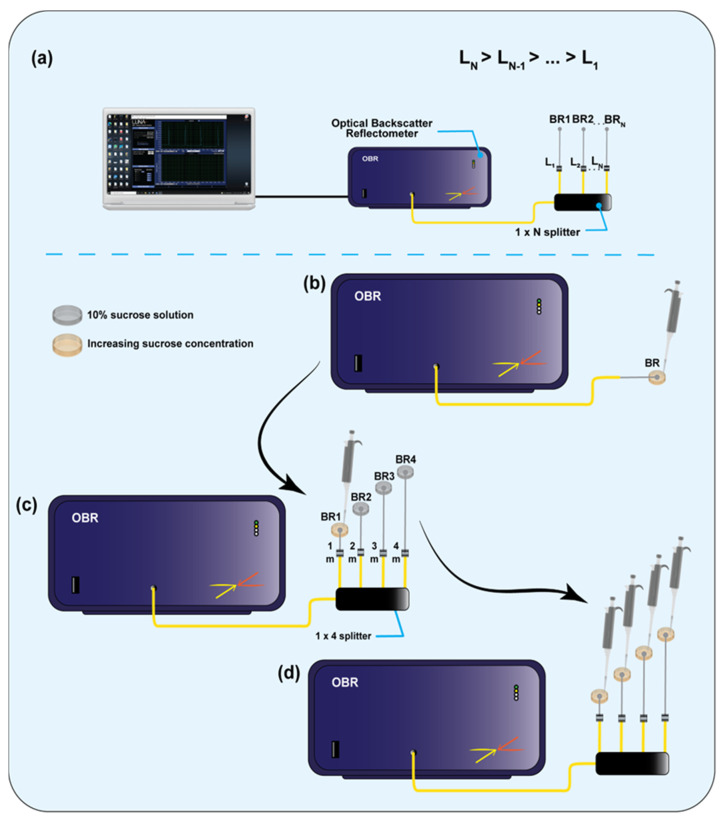
Schematics of the experimental setup. (**a**) General schematic of the proposed SDM-based multiplexing system for an arbitrary number of ball resonators (BRs), where L_i_—the length of the fiber for each i-th channel and N—total number of channels. (**b**) Schematic of individual resonator calibration setup (no network; no cross-sensitivity). (**c**) Schematic of individual resonator calibration—one at a time—within multiplex network (network; minimum cross-sensitivity). (**d**) Schematic of simultaneous calibration within multiplex network (network; cross-sensitivity).

Figure 3 shows the schematic of the proposed SDM-based multiplexing system, for an arbitrary number of BRs, as well as the schematics used for the experimental characterization. The first chart shows the generic structure of the SDM method: the OBR is connected to a 1 × N splitter for the interrogation of N channels; each BR, fabricated on the fiber tip, is connected to the analyzer through a fiber span of different length (serving as delay line), such that multiple BRs appear on the OBR in different locations and can be separately interrogated in a single scan. This generic approach has been implemented in this work using four sensors, one for each channel.

The measurement of RI change of individual BR was done by placing each of them inside the small lid with a 10% sucrose solution. The concentration was increased, starting from 1.34783 up to 1.35077 refractive indexes, by adding 40% sucrose solution dropwise. Spectral changes were measured every 20 s after signal stabilization.

With this calibration process, each sensor was calibrated for 10 RI values ranging from 1.34783 to 1.35393, measuring an RI change of approximately 6.1 × 10^−3^ RIU (refractive index units). In this short RI range, inferior to 10^−2^ RIU, we can assume a linear relationship between the BR spectral changes and the RI variation, through small-signal analysis [41].

The OBR was set with the following parameters: wavelength range of 1525.0–1610.5 nm; resolution bandwidth of 5.15 GHz; scan range of 2 cm (length of the region analyzed on the OBR for each BR sensor). Spectral data were filtered through a low-pass Butterworth filter (5th order; cut-off digital frequency 0.02).

All RI calibrations have been performed using the approach highlighted in Figure 3, using 4 sensors in the network; results could be easily extended by increasing the 1 × N splitter size. At first, sensors were calibrated independently without the SDM network, in order to verify the influence of the sensing network. Then, the sensors were all connected to the SDM network; each sensor was independently calibrated by exposing it to variations of RI, while the other sensors were held at constant RI value. Finally, all sensors were simultaneously tested by changing the RI simultaneously for each device. In this case, we can compare the sensitivity values obtained in each condition and interpedently evaluate the effects of the fiber-optic network as well as cross-talk between sensors.

### 2.4. Surface Functionalization

In order to evaluate the performances of the SDM approach for biosensing, we employed four different ball resonators, fabricated with the same method previously described and with similar sensitivity values; two ball resonators have been functionalized for CD44 detection, while the other two were functionalized for HER2. In this work, the detection of HER2 was carried by using the therapeutic anti-HER2 monoclonal antibody Trastuzumab as a receptor for the HER2 detection.

Before functionalization, BRs were cleaned of organic contaminants, and the surface was activated (hydroxylated) by incubating each BR tip in piranha solution (sulfuric acid and hydrogen peroxide at 4:1 ratio) for 15 min. BRs were then rinsed thoroughly with DI water and gently dried under the stream of nitrogen gas. Cleaned and activated BRs were then silanized by 1% APTMS dissolved in an anhydrous methanol for 20 min. After the silanization step, BRs were rinsed with methanol several times to remove unbound silane molecules. Silanized BRs were then placed inside the convection oven for 1 h at 110 °C to further cross-link the silane molecules and to remove solution residues. The surface of the silanized BR was then treated with GA diluted in PBS for 1 h. The GA concentration used for each protein detection is presented in Table 1. GA acted as a cross-linker for antibody attachment. GA-treated BRs were then rinsed with PBS several times and eventually incubated with CD44 and HER2 monoclonal antibodies at different concentrations for 1 h, according to Table 1, followed by rinsing with PBS to remove the unbound antibodies. The surface of the BR was then blocked with a blocking agent with either BSA or mPEG-amine for 30 min, with subsequent PBS rinse. All incubations were carried out at room temperature. Functionalized BRs were then used for protein detection immediately.

### 2.5. CD44 and HER2 Protein Detection

Both CD44 and HER2 proteins were spiked in human serum (diluted at 1:10 in PBS) for detection. Different protein concentrations (ranging from 1 pM up to 100 nM diluted by 10×) were dissolved in diluted serum. Each of 16 ball resonators were incubated in a certain protein concentration for 20 min at room temperature. Spectral changes were recorded using OBR at 0 min (starting point), 10 min, and every 2 min onwards (i.e., 12 min, 14 min, 16 min, 18 min, and 20 min) during the incubation period.

### 2.6. Definition and Estimation of Performance Parameters

The two main parameters that we aim at evaluating are: (1) the cross-talk, which evaluates how the changes occurring to a ball resonator affect the detection on the other sensors; (2) the spectral deformations, which measure how the spectrum of one sensor is affected by changes occurring on the other sensors [43]. The cross-talk has a direct impact on the performance of the sensor, as it shows the uncertainty of the measurement due to the presence of the other arms of the sensing network; ideally, zero cross-talk implies that the measurement of any one sensor is not affected by the changes occurring on the other sensors. Spectral deformations occurring in the system have an indirect effect: rather than directly impacting the sensitivity, changes occurring in the spectrum detune the algorithm chosen for feature tracking [35], which depends on the spectral shape. Ideally, spectra of each sensor would be maintained as identical, regardless of how the sensing network is arranged (except for slight intensity changes due to the different return losses in the splitter); however, the low finesse of BR spectra makes them vulnerable to possible changes in the spectral envelope. In order to provide a quantitative measurement of the multiplexing capability, we define performance metrics that display how the RI measurement is affected in the multiplexed network.

The first parameter that we define is the cross-talk of the *i*-th sensor onto the *j*-th sensor, labeled as *CTij* and defined as Equation (1):

(1) $$CT_{ij}=\frac{s_{i}}{s_{j}}$$

where *s_i_* and *s_j_* are the RI sensitivity values for the *i*-th sensor and *j*-th sensor, when the RI changes only for the *j*-th sensor. We report the *CT* coefficients in a matrix (4 × 4 in this work), where the elements on the main diagonal are equal to 1. The *CT* matrix is measured by changing the RI to one sensor at a time, estimating the intensity change occurring to all four sensors, and evaluating the sensitivity through a linear fit between intensity and RI changes [35].

The other element of cross-talk is due to the presence of the network; here, we aim at estimating how the sensitivity of the sensor alone (without the presence of the other sensing elements) differs from the sensitivity recorded when all sensors are connected to the network. We estimate this by computing the network cross-talk percentage coefficient, labeled *NCi* for the *i*-th sensor, which is recorded as Equation (2):

(2) $$NC_{i}=\left|\frac{s_{i}-s_{r,i}}{s_{r,i}}\right|\times 100\%$$

where *s_r,i_* is the reference sensitivity of the *i*-th sensor, measured when only one BR sensor is connected to the network. Since one of the important elements for fiber-optic biosensors is the repeatability [41], it is important to assess that the *NC* coefficients are low, in order to ensure that the sensitivity of BRs is preserved in any working condition.

Concerning the spectral deformation, we examine first the spectral differences occurring between the sensor connected to the whole sensing network and the same sensor when all the other sensors are unplugged from the network. This showcases the impact of the back reflections occurring at the other terminals of the network on the measuring BR. The metric we choose is the standard deviation of the difference between the spectra measured with and without the network. We call *S_N,i_*(*λ,n_j_*) the spectrum of the *i*-th sensor, for the *j*-th refractive index value, when all sensors are connected to the network; *S_W,i_*(*λ,n_j_*) is the spectrum of the same sensor acquired in the same RI conditions, when all the other sensors are unplugged, and *λ* is the wavelength. In Equation (3), we compute the spectral variation coefficients as:

(3) $$SV_{ij}=\sqrt{\frac{1}{L}\sum_{\lambda }{\left\{S_{N,i}\left(\lambda ,n_{j}\right)-S_{W,i}\left(\lambda ,n_{j}\right)-\frac{1}{L}\sum_{\lambda }\left[S_{N,i}\left(\lambda ,n_{j}\right)-S_{W,i}\left(\lambda ,n_{j}\right)\right]\right\}}^{2}}$$

This process allows for treating the difference between the two spectra as a signal, of length *L*, equal to the number of wavelength points of the analyzer; by subtracting the mean value (i.e., the second summation term), we remove the effect of the fluctuations of the power levels due to the different back reflections measured by the OBR, which does not distort the spectral envelope but is just an offset on the measurement. The mean spectral variation can then be computed by averaging the *SV* coefficients over all RI values (Equation (4)):

(4) $$MSV_{i}=\frac{1}{N}\sum_{j}SV_{ij}$$

where *N* is the number of RI datapoints (10 in this work). In ideal condition, the *MSV* coefficients are equal to the accuracy of the OBR detection system, which would imply that the *MSV* coefficients’ variations have no effect on the spectral detection.

The other parameter that we track, concerning the variation of the spectral waveform, is the change of the fringe visibility (FV) of the spectrum [37]; we evaluate the FV differential between the spectra, at the maximum change of the RI, which is observed when only the *i*-th sensor is exposed to the RI change and all the other sensors are in reference condition, with respect to the case when all sensors detect the maximum change of the RI. We define the normalized FV change (*FVCi*) of the *i*-th sensor as Equation (5):

(5) $$FVC_{i}=\left|\frac{FV_{N,i}-FV_{A,i}}{FV_{N,i}}\right|\times 100\%$$

where *FVN,i* is the fringe visibility for the spectrum *SN,i*, of the *i*-th sensor, and *FVA,i* is the fringe visibility for the spectrum *SA,i*, of the *i*-th sensor that is recorded when all sensors change the RI. In Equation (5), the RV is defined, as in the classical definition of interferometry [44], as the difference between maximum and minimum of the spectral fringe, normalized by the sum of the two fringe intensities.

## 3. Experimental Results

### 3.1. Multiplexing Capability and RI Detection

**Figure 4:**
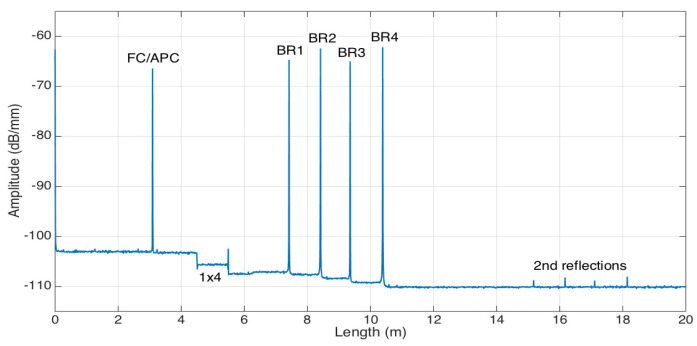
Reflectivity trace of the multiplexed BR sensing architecture; the chart reports the amplitude of the reflected signal as a function of the length, highlighting the position of the lead FC/APC connector, the 1 × 4 splitter, the 4 BR sensors, and the second-order reflections visible at >15 m distance.

The reflectivity trace measured by the OBR, with the sensing network arranged for multiplexed sensing, is shown in Figure 4. Each BR resonator appears as a sharp reflective peak with −67 to −63 dB intensity, which is comparable to the back-reflection level of a FC/APC connector. Thanks to the fiber extenders having different lengths at each channel, the reflective peaks are spaced at a distance much longer than the gauge length of the OBR, which allows for an almost perfect separation of the contribution of each sensor. From the reflection trace, we observed that the second order of reflections is visible with about an 8 m distance between each sensor, which corresponds to roughly one round-trip length of the channel fiber’s length. However, since these peaks do not overlap with the main contributions, their contribution is negligible and does not affect the measurements.

By integrating over a short window, correspondent to each reflectivity peak, and by isolating the other contributions outside of the window, we can detect the contribution to each sensor separately; we repeated this process by changing the RI for each individual sensor, in order to evaluate the multiplexing capability.

**Figure 5:**
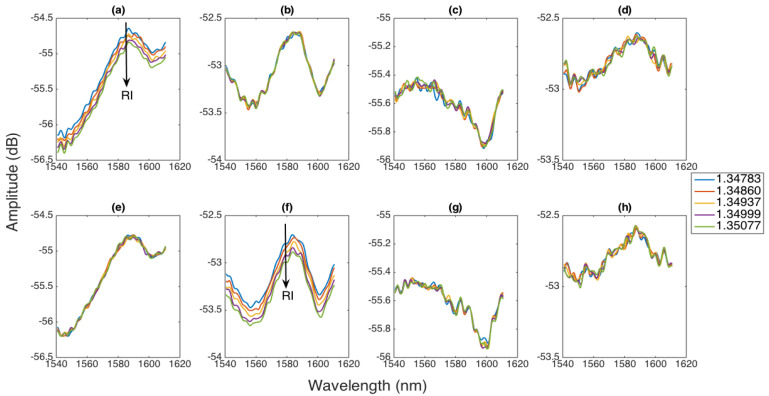
Evaluation of the multiplexing capability of the proposed approach. The chart proposes the spectra of each BR sensor on each column (left to right: BR1, BR2, BR3, and BR4) as the RI changes from 1.34783 to 1.35077. First row: spectra of (**a**) BR1, (**b**) BR2, (**c**) BR3, and (**d**) BR4 sensors when the BR1 sensor is exposed to a RI change, while the other three sensors are held at the reference RI. Second row: spectra of (**e**) BR1, (**f**) BR2, (**g**) BR3, and (**h**) BR4 sensors when the BR2 sensor is exposed to a RI change, while the other three sensors are held at the reference RI.

The capability of the multiplexing setup is highlighted in Figure 5, where we report the spectra of all the ball resonators when the RI changes for one sensor, while the other sensors are held at constant RI; for the sake of brevity, we report the behaviors obtained by varying the RI on BR1 (first row) and BR2 (second row), but a similar result is obtained for all the sensors.

We observe that, on the spectral pattern, the multiplexing method works with great accuracy. All the spectra of the ball resonators, in agreement with [35], show shallow spectral fringes due to the weak interferometric structure. When the RI changes in BR1, as in Figure 5a, we observe that the spectrum decays by approximately 0.2 dB, as the RI increases by 0.003 RIU. The sensitivity can either be estimated by measuring the spectral intensity or the wavelength shift of each fringe; the first method is more independent of the specific pattern of the spectral fringes, and, therefore, we will estimate the sensitivity of the sensors as the intensity change as a function of the RI change (in dB/RIU, which makes it comparable with tilted FBGs [3], U-bent fibers [45], or similar sensing approaches).

Thanks to the multiplexing setup, the other sensors, BR2, BR3, and BR4, are unaffected by the changes occurring to BR1; in fact, their spectrum does not change, as they are held at constant RI, and the changes occurring on the neighbor sensors have no effect on the other ones, proving the effectiveness of multiplexing. As a proof, Figure 5b–d show that each spectrum of BR2, BR3, and BR4 remains constant with respect to the RI changes occurring to the first sensor.

A similar behavior is shown in Figure 5e–h for changes occurring to BR2. In this chart, we observe that the RI change occurring on the second sensor can be recorded as an intensity drop in the spectrum, whereas the other sensors do not observe a detectable spectral change.

**Figure 6:**
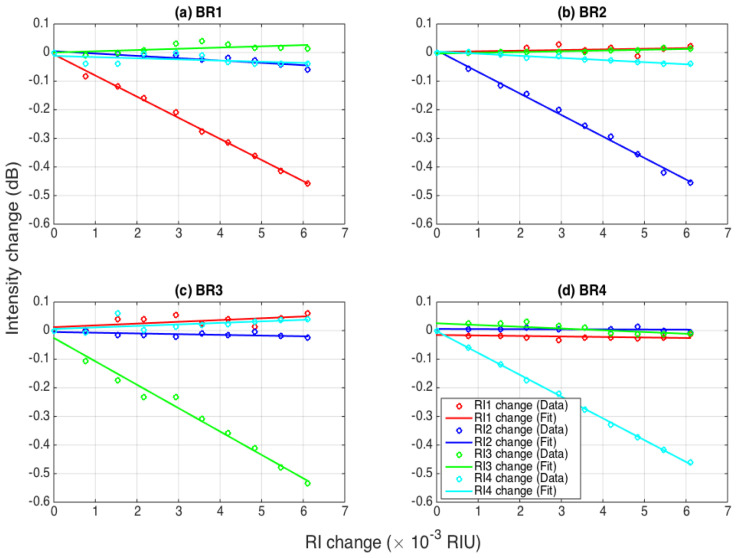
Evaluation of the intensity change for each ball resonator sensor as a function of the RI, within the multiplexed sensing network. The charts show the intensity change for each BR sensor: (**a**) BR1, (**b**) BR2, (**c**) BR3, and (**d**) BR4. Each curve shows the response of the sensor when the RI changes (from 0 to 6.1 × 10^−3^ RIU) for one sensor, while the other RI values remain constant: red = BR1 RI changes; blue = BR2 RI changes; green = BR3 RI changes; cyan = BR4 RI changes. Measured data are reported as circles; linear fits are reported as solid lines.

By tracking the intensity change for each ball resonator in the same way, as a function of the RI change, we can observe how sensitive each BR sensor is to the RI and the effect of cross-sensitivities; the resulting data are shown in Figure 6, where the intensity change as a function of RI is reported for each sensor and for each RI change.

As expected, each sensor shows a clear sensitivity to the RI change, as the intensity drops by 0.45–0.53 dB, for an RI increase of 0.61 × 10^−3^ RIU. On the other side, the intensity of each BR resonator is not significantly affected by the changes of the RI occurring to the other sensors connected to the network. Despite the small RI changes, the sensitivity is clear and can be estimated through a linear fit that also allows for estimating the cross-sensitivity elements.

### 3.2. Evaluation of Sensitivity and Cross-Talk Figures

**Figure 7:**
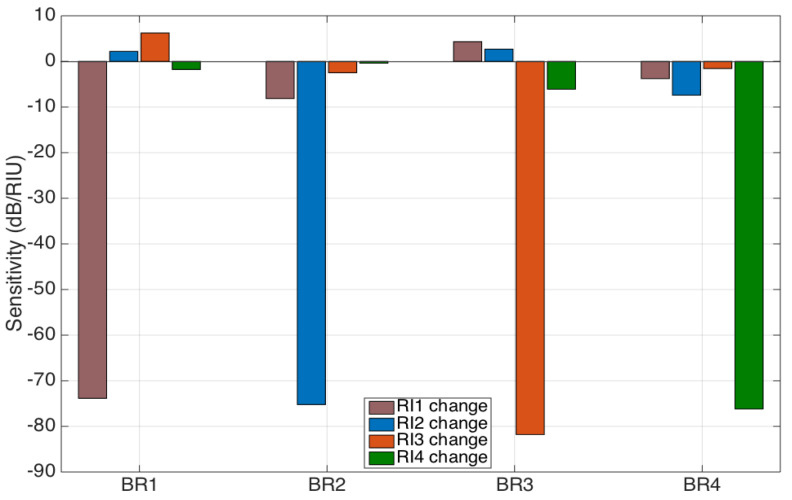
Sensitivity recorded for each BR sensor (left to right: BR1, BR2, BR3, and BR4) experienced when the RI changes for one sensor (saddle: BR1 changes; blue: BR2 changes; orange: BR3 changes; green BR4 changes). All sensitivity values are estimated as the slope of the linear fits observed in Figure 6.

The RI sensitivity for each sensor and for each cross-talk element is shown in Figure 7, which reports the 16 sensitivity values recorded for each sensor and for each RI change. The BR sensors developed for this work have similar values of sensitivity: the obtained values are −73.8 dB/RIU (BR1), −75.2 dB/RIU (BR2), −81.8 dB (BR3), and −76.2 dB/RIU (BR4), respectively. These values are in line with previous values of BR sensitivities, usually within 70–100 dB/RIU [42].

Cross-sensitivities are also experienced, and, as expected, they have a much smaller value, with recorded values of cross-sensitivities that range from −0.4 dB/RIU to −8.1 dB/RIU, with some values being toward the positive sign (which is possibly due to the different formats of the spectral features typical in ball resonators).

We can provide a quantitative measurement of the cross-talk, by displaying the *CT* parameters defined in Equation (6), which are directly inferred from Figure 7:

(6) $$CT=\left[\begin{array}{cccc} 1.000 & 0.110 & -0.058 & 0.053\\ -0.029 & 1.000 & -0.036 & 0.099\\ -0.076 & 0.030 & 1.000 & 0.014\\ 0.023 & 0.005 & 0.080 & 1.000 \end{array}\right]$$

where we can observe that the worst cross-talk effect is 11.0% (*CT*12). The determinant of the *CT* matrix is equal to 0.998, which is very close to the ideal case (identity matrix). However, a more conservative evaluation of the influence of the cross-talk in a measurement is to evaluate the impact of a worst-case scenario, i.e., the sum of the moduli of all the elements on each row outside of the main diagonal, which display, in the worst possible case, the impact of an RI change occurring on all the other sensor on the diagonal (main) sensor. This factor ranges from 10.8% for the BR4 sensor up to 22.1% for the BR1 sensor and represents the worst-case scenario for the cross-sensitivity.

If we consider the average case, we can consider that the cross-talk impacts for a relative uncertainty of 5–11%, which can possibly be mitigated by measuring a wavelength shift rather than an intensity change, as this is a more robust method for tracking [38]. Considering that the BR resonators have great sensitivity for the measurement of small RI changes, an additional uncertainty in the range of ~5–11% for the measurement of RI values in the order of 10^−4^ RIU can be considered an acceptable impairment, given the benefit of multiplexed networks; in biosensors, this uncertainty is similar or inferior to the typical specificity errors [41].

**Figure 8:**
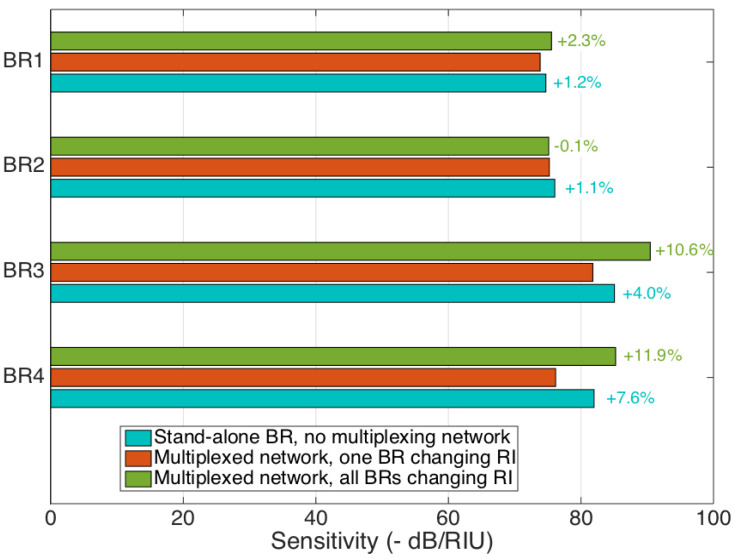
Comparison of the sensitivity values for each ball resonator, recorded in different working conditions: stand-alone ball resonator, without the splitter and the multiplexing network (cyan); all BRs connected to the multiplexed network, with only one BR changing the RI (red, reference); all BRs connected to the multiplexed network, with all RIs simultaneously changed (green).

In Figure 8, we compare the sensitivity of each BR sensor to the RI recorded in each working condition, as illustrated in Figure 2. The reference-curve-sensitivity value, for multiplexing, is observed by varying the RI for each sensor separately and maintaining the other BRs at a reference RI. The first comparison is with the sensitivity recorded for each sensor, which is measured individually for the same RI values and unplugged from the network. This measurement allows for evaluating the effect of the network, which induces additional losses and the multiple reflections that might affect the frequency-domain-processed data by the OBR [39]. We observe that the sensitivity varies, in these conditions, by a factor included within 1.1% and 7.6% for the sensors; as in Section 4, this can be accounted for by the *NC* metric for the four sensors (Equation (7)):

(7) $$NC=\left[\begin{array}{cccc} 1.2\% & 1.1\% & 4.0\% & 7.6\% \end{array}\right]$$

with an average deviation of 3.5%.

On the other hand, the sensitivity observed when all sensors (including the ones not under test) are changing the RI varies from −0.1% to 11.9%, at 6.2% on average. Here, a higher deviation is expected to be found, as this takes into account the cross-talk rather than the network effect. However, we can conclude that the variations of the sensitivities recorded within the different working conditions are well within the repeatability observed for optical-fiber biosensors [41], which confirms that the impairments of the multiplexing network, despite being quantifiable, do not compromise the quality of the RI measurement.

### 3.3. Evaluation of Spectral-Variation Metrics

**Figure 9:**
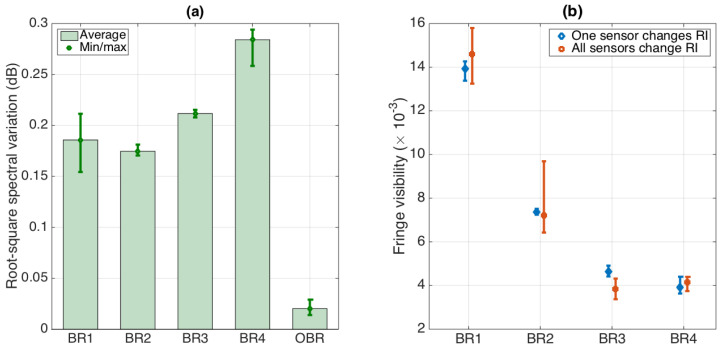
Evaluation of spectral variation metrics for the multiplexed ball resonators. (**a**) Root-square spectral variation between multiplexed sensor and stand-alone sensor, reported for each BR sensor and compared to the OBR limit. The vertical bars show the average over the RI values, and error bars show the minimum/maximum range. (**b**) Fringe visibility of the spectra of each BR sensor, reported when each sensor varies the RI (blue), and when all sensors are subjected to RI change (red). Error bars report the minimum/maximum values; datapoints report the mean value.

An evaluation of the spectral variation metrics, namely the root-square spectral variation expressed in Equation (3) and the fringe visibility obtained in each working condition, is shown in Figure 9.

The first chart shows the root-square spectral variation, showing both the average and minimum/maximum values for each BR sensor and comparing with the root-mean-square error of the spectral estimate or the OBR (estimated for 10 flat spectra processed through the same digital low-pass filter, to provide a fair comparison). The *MSV* coefficients have the following values (Equation (8)):

(8) $$MSV=\left[\begin{array}{cccc} 0.186\;\mathrm{db} & 0.175\;\mathrm{db} & 0.212\;\mathrm{db} & 0.284\;\mathrm{db} \end{array}\right]$$

while the OBR limit is 0.020 dB, about one order of magnitude inferior (which would lead to an RI detection limit of approximately 2.8 × 10^−4^ RIU). Considering that the sensors, as shown in Figure 5, have spectral variations that fluctuate by ~2 dB, the *MSV* plays a role in distorting the spectral fluctuations. These spectral changes are often common in ball resonators, though they might be emphasized when transitioning from a single-sensor configuration to a multi-sensor network. The error bars show the variability of the results, which provides an estimate on how noisy the detection process is.

The second chart shows the FV recorded for each sensor, comparing the two working cases: only one sensor changing the RI (reference case) and all sensors changing the RI (which is affected by the cross-talk). The FV is a metric that is noise-sensitive and, therefore, shows a high variability, which is different for each sensor, as it is included within 0.0038 (BR3) and 0.014 (BR1): typical values for ball resonators [37] that denote a weak interference fringe.

The normalized FV change is measured as Equation (9):

(9) $$FVC=\left[\begin{array}{cccc} 4.8\% & 1.8\% & 16.9\% & 6.2\% \end{array}\right]$$

for the four ball resonators. Consistently, as the FV is a noisy metric, we observe the largest variations for the sensors that have weaker interference fringes, with the maximum observed for BR3, which is much inferior for the sensors characterized by a higher FV. Overall, the average FV change is 7.4%, which does not provide much detuning from the spectral peaks. Preserving the FV is important for the feature extraction algorithm [37], which is usually based on spectral peak/valley identification and the tracking of normalized spectra; therefore, this estimate provides a sufficient quality for multiplexing.

### 3.4. Detection of Cancer Biomarkers

In order to assess the capability of the SDM method for multiplex biosensors, we performed a simultaneous interrogation of four different BR sensors, functionalizing two of them for each cancer biomarker under analysis (CD44 and HER2, respectively). In order to maximize the sensitivity, as in [37], we report the traces acquired for the S-polarized light (S = perpendicular polarization, referring to the OBR laser). The BR sensors reported in this section are from a different batch than the ones previously reported, having a similar fabrication method but functionalized for biomarker detection.

**Figure 10:**
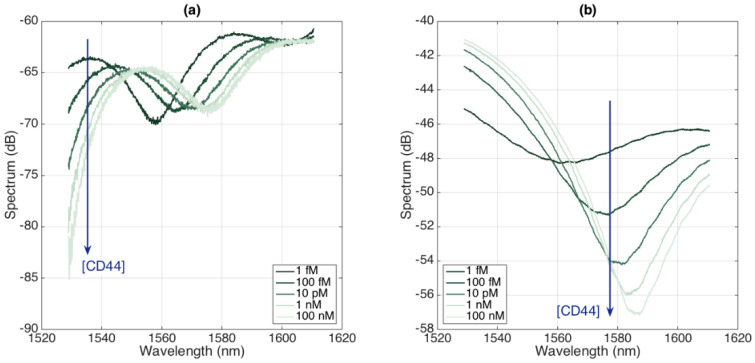
Spectra of BR sensors functionalized for CD44 detection, for different concentrations ranging from 1 fM to 100 nM. (**a**) First sensor; (**b**) second sensor.

**Figure 11:**
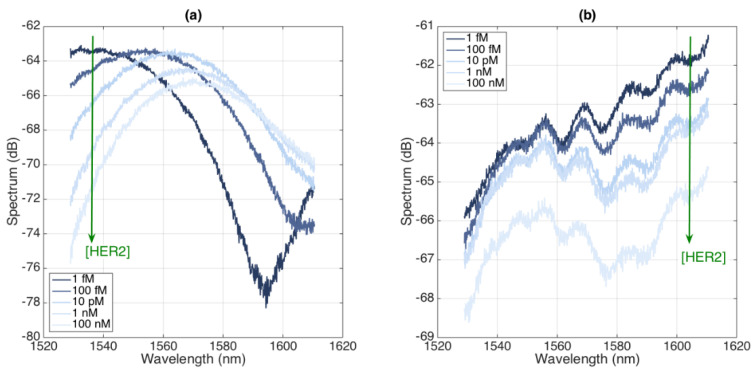
Spectra of BR sensors functionalized for HER2 detection, for different concentrations ranging from 1 fM to 100 nM. (**a**) First sensor; (**b**) second sensor.

The spectra of the BR sensors are reported in Figure 10 (CD44) and Figure 11 (HER2), each reporting the spectra for two sensors. We notice that the spectral fingerprints appear, similarly to those of the prior sensors, as almost random envelopes with intensity changes occurring for each increment of the concentration of the analyte.

**Figure 12:**
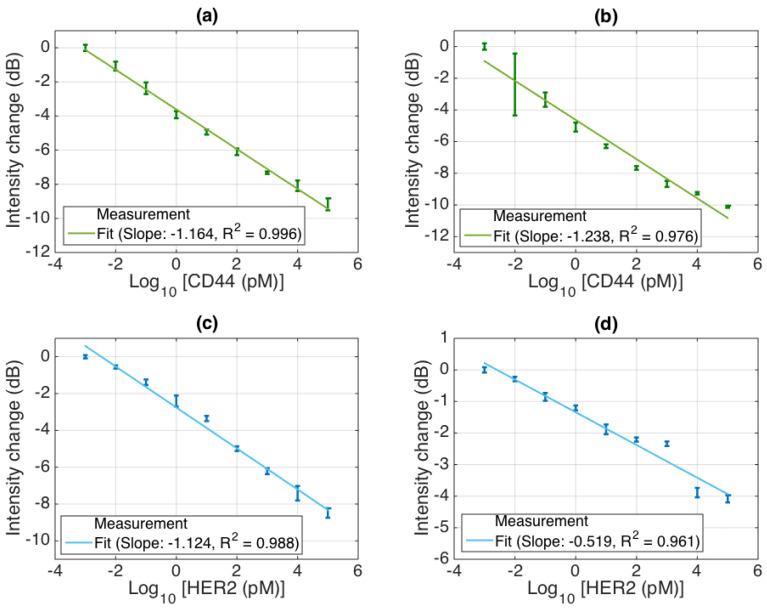
Detection of cancer biomarkers with BR sensors, using a simultaneous four-sensor network. Each chart reports the experimental data, sampling the concentrations from 1 fM to 100 nM with a 10× increment; error bars report ± standard deviation of 6 consecutive measurements, acquired after 10 min stabilization and sampled at each 2 min interval. Solid lines report the log-linear fit; the slope reports the intensity change (in dB units) recorded for each 10× increment of concentration. (**a**) First CD44 sensor; (**b**) second CD44 sensor; (**c**) first HER2 sensor; (**d**) second HER2 sensor.

From a spectral fingerprint, by interrogating one spectral feature that exhibits a sensitivity to the protein concentration (highlighted with an arrow in Figure 10 and Figure 11), we can perform the detection of cancer biomarkers; the results for each sensor are reported in Figure 12, recording both the experimental data (statistics accumulated over six measurements at a 2 min interval between each sampling) and a log-linear fit. The CD44 sensors have sensitivity ratings of 1.164 and 1.238 dB for each 10× concentration increase, while the HER2 biosensors reported sensitivity ratings of 1.124 and 0.519 dB for the same 10× concentration rise, each achieved with a coefficient of determination of R^2^ > 0.96.

## 4. Discussion

Multiplexing is key to enable the advanced sensing features envisioned by tomorrow’s label-free biosensors [46]; therefore, it is essential to provide sensors that are suitable for multiplexing while leading to a rapid fabrication. Undoubtedly, the SDM-based multiplexed BR sensors accomplish both tasks: the manufacturing of the ball resonators is rapid, as it applies the techniques used in diffractive lenses fabrication to single-mode fibers with a smaller core size [47], and the multiplexing is guaranteed as the experimental data show that the contribution of each sensor can be separated from the contributions of the other ones. The main weakness is that the sensors induce spectra with low fringes, which are harder to detect and are more vulnerable to noise.

When compared to the TDM and WDM multiplexing methods, the SDM applied to ball resonators appears more exposed to the surrounding conditions. In TDM, the channels are physically separate, and, therefore, only one channel is consistently switched on. WDM is very common for FBGs, which have reflectivity values that are confined to a very small bandwidth (<1 nm), allowing for the strong isolation of each wavelength window; the reflectivity of an FBG outside of the main spectral lobe is substantially zero.

Theoretically, the SDM-based multiplexing method applied to the OBR interrogator would also work, as the location of each sensing peak can be isolated in the instrument and separated from the surrounding contributions, while second- or higher-order reflections are clearly out of the window of analysis. However, the local spectra of OBR sensors are evaluated through the inverse Fourier transform of the reflected waves, as shown by Froggatt and Moore [39], which implies that cross-talk effects might affect spectral estimates. This effect is minimal when the OBR interrogates high-reflectivity devices such as FBGs [48], as their reflectivity is so high that it is not affected by the surrounding conditions; it is also marginal when interrogating a single BR sensor, as the scattered power of an SMF fiber is several orders of magnitude inferior to the reflectivity levels of a BR. However, in a multiplexed network, where multiple sensors with similar reflectivity are present, spurious effects can affect the measurements.

In addition, biosensors and RI sensors are inherently measuring small changes of RI, as the key benefit of optical biosensors lies in their top-tier performance figures (compared, for example, to those of transistors or electrochemical impedance spectroscopy [49], particularly regarding the limit of detection). Therefore, it is important to evaluate the results proposed in this experimental validation in light of this specific context.

The cross-talk reported in Figure 6, Figure 7 and Figure 8 is not a negligible factor, but it is close to the standard repeatability and specificity observed in biosensors [41], considering that the calibration is done on RI steps of 6.8 × 10^−4^ RIU, and, therefore, cross-sensitivity terms might be overestimated due to the impact of noise. The fact that the change of sensitivity appears to affect more sensors characterized by lower FV is consistent with the detection method, as such sensors are harder to track in terms of feature extraction and analysis. A solution to this problem would be found by consistently manufacturing BR sensors having a higher fringe visibility, which is often the case with resonators having a smaller diameter [50].

On the other hand, the spectra observed under different measurement conditions maintain a very close fringe visibility, but they tend to slightly differ in shape. This effect is inherent to the OBR interrogation, since with multiple sensors the reflectivity to the detector is higher, which might slightly affect the Fourier transform that is at the base of the OBR processing. A possible solution to this drawback is to improve the feature tracking, possibly by employing methods that work on the whole spectrum rather than on a single feature, or to use an FBG interrogator or similar analyzer that has different hardware [10].

Overall, the performances obtained experimentally are, however, consistent with the performance and downsides of a BR sensor and in line with most of the latest biosensors in terms of sensitivity and repeatability. By enabling a multiplexing with simultaneous scan, this typology of sensors can add a significant dimensionality in modern RI sensing and biosensing.

## 5. Conclusions

In this work, we report and experimentally demonstrate a spatial-division multiplexing method for the multiplexing of fiber-optic ball resonators. BRs are RI sensors characterized by a high sensitivity, ease of manufacturing, and low-fringe quasi-random spectrum. The SDM method, based on a splitter and a set of fiber extenders, allows for separating the contribution of each sensor, allowing for multi-sensor detection with a single scan. The method has been validated on an OBR interrogator, with a 1 × 4 configuration tested in different working conditions, which encompass the use of BRs as refractometers as well as for biosensors detecting multiple proteins. The cross-talk and spectral variation figures have been measured, obtaining values compatible with the performance and repeatability of fiber-optic biosensors. The experimental results show that in different operative conditions the sensitivity changes by up to 11.9%, while the cross-talk is up to 11.0%; the variation in terms of fringe visibility is 7.4% on average. These data show that the SDM approach allows for extending the detection from one to multiple ball resonators, enabling biosensing multiplexing, despite the sensors sharing the same operative bandwidth.

Future work will involve implementing the SDM approach for multiplexed biosensors, in order to simultaneously track a plurality of biomarkers, and approaching the levels of complexity currently implemented in technologies such as lab-on-chip [51,52] and immunoassays [53,54], while maintaining the advantageous features of optical-fiber biosensors such as real-time label-free detection, in situ operation, and excellent performance at low concentration limits.

## Figures and Tables

**Table 1:** Conditions used to functionalize BRs.

	GA Dissolved in PBS	Antibody Concentration	Blocking	References
For CD44 detection	25%	4 µg/mL and 2 µg/mL	10% mPEG-amine	[42]
For HER2 detection	2.50%	20 µg/mL and 8 µg/mL	1% BSA	[28]

## Data Availability

Not applicable.
